# Low-frequency quantum oscillations in LaRhIn_5_: Dirac point or nodal line?

**DOI:** 10.1038/s41467-023-37692-6

**Published:** 2023-04-13

**Authors:** G. P. Mikitik, Yu. V. Sharlai

**Affiliations:** 1 B. Verkin Institute for Low Temperature Physics and Engineering of National Academy of Sciences of Ukraine, Kharkiv, 61103 Ukraine; 2 Institute of Low Temperature and Structure Research, Polish Academy of Sciences, 50-422 Wrocław, Poland

**Keywords:** Topological matter, Electronic properties and materials

**arising from** C. Guo et al. *Nature Communications* 10.1038/s41467-021-26450-1 (2021)

In the recent paper^1^, a new method based on measuring a temperature correction to a quantum-oscillation frequency was proposed to study an energy-band dispersion of charge carriers in small Fermi surface (FS) pockets of crystals. To illustrate their approach, the authors of ref. ^1^ applied it to a number of materials and, in particular, to the multiband metal LaRhIn_5_, which, apart from high-frequency oscillations associated with a large FS, also exhibits the oscillations with the low-frequency *F* ≈ 7 T. Although the method of ref. ^1^ really detects charge carriers with linear dispersion, it does not distinguish between the carriers near a Dirac point and near a nodal line, since all such quasiparticles disperse linearly. Here we ask what is the nature of the carriers associated with the frequency *F* in LaRhIn_5_ and call attention to the puzzling origin of this frequency.

Many years ago^2^, we argued that the 7 T frequency is due to the minimal cross section of an FS surrounding a nodal line in LaRhIn_5_, whereas the authors of  ref. ^1^ now relate this frequency with a cross section of an FS pocket enclosing a Dirac point. Below we show that the main experimental result of ref. ^1^ does not contradict our assumption of the nodal line in LaRhIn_5_. The degeneracy of two bands *ε*_c_(**p**) and *ε*_v_(**p**) along a nodal line, strictly speaking, occurs in LaRhIn_5_ only when neglecting a weak spin–orbit interaction. Consider now these bands in the vicinity of some point **p**_0_ of the line, taking into account this interaction^3, 4^ (Fig. 1),

1 $${\varepsilon }_{{{{{{{{\rm{c,v}}}}}}}}}({{{{{{{\bf{p}}}}}}}})\,=\,{\varepsilon }_{{{{{{{{\rm{d}}}}}}}}}+{{{{{{{\bf{a}}}}}}}}{{{{{{{\bf{p}}}}}}}}+b{p}_{z}^{2}\pm \,\,\sqrt{{{{\Delta }}}^{2}\,+\,{({v}_{x}{p}_{x})}^{2}\,+\,{({v}_{y}{p}_{y})}^{2}}$$

where Δ ≡ Δ(**p**_0_) is half of the spin–orbit gap at the point **p**_0_, *ε*_d_ is the band-degeneracy energy at this point in the absence of the spin–orbit coupling (i.e., when Δ = 0), *v*_*x*_, *v*_*y*_, **a** = (*a*_*x*_, *a*_*y*_, *a*_*z*_), and *b* are constant parameters, the quasimomentum **p** is measured from **p**_0_, the *p*_*z*_ axis coincides with the tangent to the band-contact line at this point, and the *p*_*x*_, *p*_*y*_ axes are chosen in such a way that the quadratic form under the square root is diagonal. Let the magnetic field be directed along *p*_*z*_. The cross section of an FS surrounding the nodal line by the plane *p*_*z*_ = constant is a closed curve (an ellipse) only if ${\tilde{a}}_{\perp }^{2}\equiv {({a}_{x}/{v}_{x})}^{2}+{({a}_{y}/{v}_{y})}^{2} < 1$. The parameter ${\tilde{a}}_{\perp }$ characterizes the tilt of the spectrum at constant *p*_*z*_, and ${\tilde{a}}_{\perp } \, \ne \, 0$ for all real situations. If the cross-sectional area at *p*_*z*_ = 0 is extremal with respect to *p*_*z*_, then *a*_*z*_ = 0, and the term $b{p}_{z}^{2}$ is taken into account in Eq. (1).

**Fig. 1:**
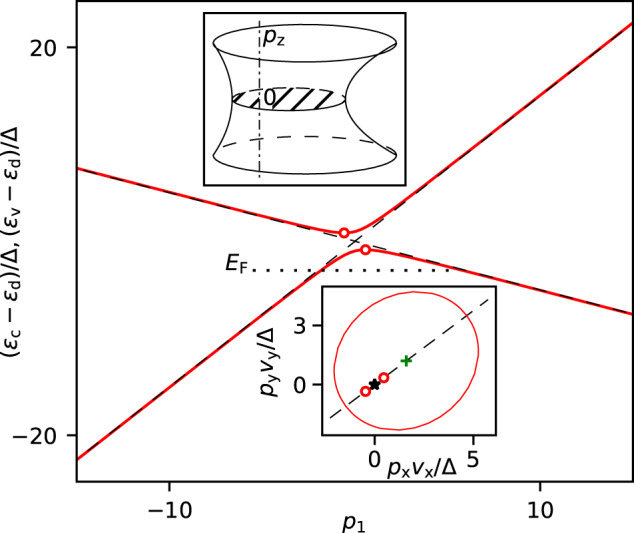
The energy bands *ε*_c_(**p**) and *ε*_v_(**p**), Eq. (1), in the vicinity of their nodal line in the plane *p*_*z*_ = 0 perpendicular to the line. The red solid and black dashed lines show the bands with and without the spin–orbit interaction, respectively. The red circles mark the minimum of *ε*_c_(**p**) and the maximum of *ε*_v_(**p**) in the plane. The minimal indirect gap $2{{{\Delta }}}_{\min }=2{{\Delta }}{(1-{\tilde{a}}_{\perp }^{2})}^{1/2}$ determined by these two points is less than 2Δ, the spin–orbit gap at **p** = 0. Here ${p}_{1}\equiv ({a}_{x}{p}_{x}+{a}_{y}{p}_{y})/({\tilde{a}}_{\perp }{{\Delta }})$ is the dimensionless quasimomentum measured along the vector $({\tilde{a}}_{x},{\tilde{a}}_{y})$ in the plane with the coordinates *p*_*x*_*v*_*x*_/Δ and *p*_*y*_*v*_*y*_/Δ; ${\tilde{a}}_{i}\equiv {a}_{i}/{v}_{i}$, and ${\tilde{a}}_{\perp }\equiv {({\tilde{a}}_{x}^{2}+{\tilde{a}}_{y}^{2})}^{1/2}$. The dotted line indicates the Fermi level *E*_F_. Upper inset: The Fermi surface enclosing the nodal line (the dash-dotted line) at (*E*_F_ − *ε*_d_)*b* < 0. Lower inset: The cross section (ellipse) of the Fermi surface on the plane *p*_*z*_ = 0. The black dashed line marks the direction along which the bands are shown in the main panel. The black asterisk and green cross mark the point **p** = 0 and the center of the ellipse, respectively.

The temperature dependence of the quantum-oscillation frequency *F* looks as follows^1^:

2 $$F({E}_{{{{{{{{\rm{F}}}}}}}}},T)={F}_{0}-\theta \frac{{(\pi {k}_{{{{{{{{\rm{B}}}}}}}}}T)}^{2}}{{F}_{0}{\beta }^{2}}$$

where *E*_F_ is the Fermi energy, *F*_0_ is the frequency of these oscillations at zero temperature, *β* = *eℏ*/2*m*_c_, *m*_c_ is the cyclotron mass, and *θ* = 1/16 for a band with linear dispersion. With Eq. (1) and the formulas of ref. ^1^ for *θ*, we arrive at

3 $$\theta=\frac{1}{16}\left(1-\frac{{{{\Delta }}}_{\min }^{2}}{{({E}_{{{{{{{{\rm{F}}}}}}}}}-{\varepsilon }_{{{{{{{{\rm{d}}}}}}}}})}^{2}}\right)$$

where ${{{\Delta }}}_{\min }={{\Delta }}{(1-{\tilde{a}}_{\perp }^{2})}^{1/2}$ is the minimal indirect half-gap in the plane *p*_*z*_ = 0 (Fig. 1). In ref. ^1^, the simplified spectrum with ${a}_{x}={a}_{y}={\tilde{a}}_{\perp }=0$ was implied, and it was concluded that the value *θ* = 1/16, which was experimentally obtained for LaRhIn_5_, can occur only if the direct spin–orbit gap is perturbatively small, ${{{\Delta }}}^{2}/{({E}_{{{{{{{{\rm{F}}}}}}}}}-{\varepsilon }_{{{{{{{{\rm{d}}}}}}}}})}^{2} \, \ll \, 1$. On the other hand, the band-structure calculations^1^ revealed that this ratio, in general, is not very small for LaRhIn_5_, and Guo et al. ascribed the frequency *F* to a cross section passing through a Dirac point (when Δ ≡ 0), excluding the case of the nodal line from their consideration. However, Eq. (3) demonstrates that the perturbatively small Δ is not necessary to obtain *θ* ≈ 1/16. It is sufficient if only the indirect spin–orbit gap in the plane of the extremal cross section of the FS is small, ${{{\Delta }}}_{\min }^{2}/{({E}_{{{{{{{{\rm{F}}}}}}}}}-{\varepsilon }_{{{{{{{{\rm{d}}}}}}}}})}^{2} \, \ll \, 1$, and so a nodal line can lead to *θ* ≈ 1/16 even though the spin–orbit coupling is not perturbatively weak in LnRhIn_5_.

It was shown earlier^2^ that the experimental dependence^5^ of the longitudinal magnetization of LaRhIn_5_ on the magnetic induction *B* can be explained if a nodal line penetrates the minimal cross section of the FS in this material (Fig. 2). Let us now discuss the case of an FS pocket enclosing the Dirac point assumed by Guo et al.^1^. A formula for the magnetization of such a pocket with a linear dispersion of its charge carriers was derived many years ago^6^, and a convenient representation^4, 7^ of this formula reads:

4 $$M=CFg(u)$$

where the positive coefficient *C* depends on Dirac-spectrum parameters, *u* ≡ *F*/*B*, and *g*(*u*) is a universal function independent of any parameters. The magnetization calculated with Eq. (4) at *F* = 7 T is shown in Fig. 2. The value of *C* is chosen in such a way that the calculated amplitude of the oscillations agrees with the experimental data. Figure 2 reveals a qualitative disagreement between this theoretical curve and the data. The theoretical curve (which is the same for electron and hole Dirac pockets) exhibits sharp peaks, whereas the experimental data reveal sharp troughs. Moreover, the behavior of the magnetization at *B* > *F* essentially deviates from the experimental dependence *M*(*B*). In other words, the assumption that the low-frequency oscillations are determined by a Dirac pocket is incompatible with the data^5^ on the magnetization of LaRhIn_5_. On the other hand, Supplementary Fig. 4 of ref. ^1^ shows that the frequency *F* = 7 T of the Shubnikov–de Haas oscillations is practically independent of the direction of the magnetic field. This result is inconsistent with the nodal-line assumption, which leads to $F(\psi ) \sim 1/\cos \psi$ where *ψ* is the angle between *B* and the line, and so the authors of ref. ^1^ assumed the existence of the Dirac point. Thus, at present, there is no self-consistent explanation of the 7 T oscillations in LaRhIn_5_.

**Fig. 2:**
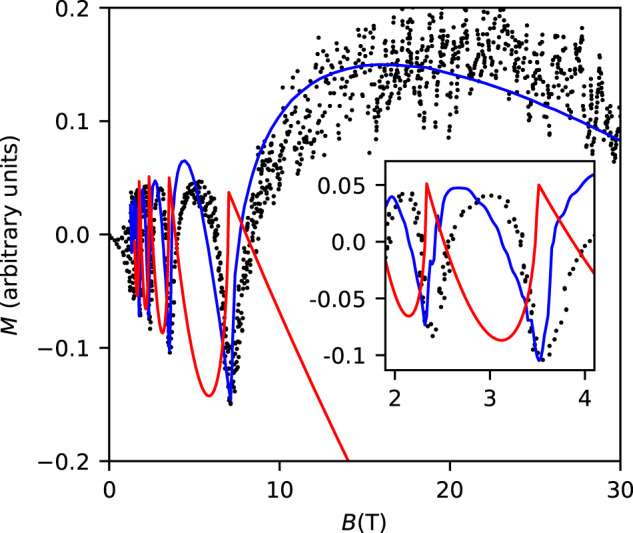
Magnetization of LaRhIn_5_. The dots are the experimental data^5^, the blue line shows the magnetization^2^ produced by the nodal line in Fig. 1, whereas the red line is the magnetization of the Dirac pocket, with the background term *χ*_0_*B* = −0.7*Cg*(1)*B* being added to Eq. (4). This term is determined by the charge carriers that are far away from the point **p** = 0^2^. The inset is a zoom into the region 2 ≤ *B* ≤ 4 T.

The Fermi surface near the nodal line (upper inset in Fig. 1) and the appropriate *M*(*B*) (blue line in Fig. 2) are shown for (*E*_F_ − *ε*_d_)*b* < 0. If the small difference *E*_F_ − *ε*_d_ changes its sign, the anisotropy of the frequency *F*(*ψ*) noticeably decreases, and *M*(*B*) resembles the red curve in Fig. 2^2^. Thus, the previously published data^1, 5^ look as if they were obtained on crystals with slightly different *E*_F_ but with practically equal ∣*E*_F_ − *ε*_d_∣. This hypothesis can be verified, measuring both the Shubnikov–de Haas oscillations and the longitudinal magnetization *M*(*B*) in one and the same sample. Then, in a sample with *M*(*B*) like in ref. ^5^, the dependence *F*(*ψ*) has to be strongly anisotropic, whereas in a sample with a weak dependence *F*(*ψ*), *M*(*B*) cannot exhibit the sharp troughs visible in the oscillations in Fig. 2.

## Data Availability

Data sharing not applicable to this article as no datasets were generated during the current study.
